# Comparative transcriptome and flavonoids components analysis reveal the structural genes responsible for the yellow seed coat color of *Brassica rapa* L.

**DOI:** 10.7717/peerj.10770

**Published:** 2021-03-04

**Authors:** Yanjing Ren, Ning Zhang, Ru Li, Xiaomin Ma, Lugang Zhang

**Affiliations:** 1 Academy of Agriculture and Forestry Sciences, Qinghai University, Xining, China; 2 Qinghai Key Laboratory of Vegetable Genetics and Physiology, Xining, China; 3 State Key Laboratory of Crop Stress Biology for Arid Area, Northwest A&F University, Yangling, China; 4 State Key Laboratory of Vegetable Germplasm Innovation, Tianjin, China

**Keywords:** *Brassica rapa*, Transcriptome, LC-MS/MS, Seed coat color, Flavonoid biosynthesis pathway

## Abstract

**Background:**

Seed coat color is an important horticultural trait in *Brassica* crops, which is divided into two categories: brown/black and yellow. Seeds with yellow seed coat color have higher oil quality, higher protein content and lower fiber content. Yellow seed coat color is therefore considered a desirable trait in hybrid breeding of *Brassica rapa, Brassica juncea* and *Brassica napus*.

**Methods:**

Comprehensive analysis of the abundance transcripts for seed coat color at three development stages by RNA-sequencing (RNA-seq) and corresponding flavonoids compounds by liquid chromatography-tandem mass spectrometry (LC-MS/MS) were carried out in *B. rapa*.

**Results:**

We identified 41,286 unigenes with 4,989 differentially expressed genes between brown seeds (B147) and yellow seeds (B80) at the same development stage. Kyoto Encyclopedia of Genes and Genomes enrichment analysis identified 19 unigenes associated with the phenylpropanoid, flavonoid, flavone and flavonol biosynthetic pathways as involved in seed coat color formation. Interestingly, expression levels of early biosynthetic genes (*BrCHS*, *BrCHI*, *BrF3H*, *BrF3’H* and *BrFLS*) in the flavonoid biosynthetic pathway were down-regulated while late biosynthetic genes (*BrDFR*, *BrLDOX* and *BrBAN*) were hardly or not expressed in seeds of B80. At the same time, *BrTT8* and *BrMYB5* were down-regulated in B80. Results of LC-MS also showed that epicatechin was not detected in seeds of B80. We validated the accuracy of our RNA-seq data by RT-qPCR of nine critical genes. Epicatechin was not detected in seeds of B80 by LC-MS/MS.

**Conclusions:**

The expression levels of flavonoid biosynthetic pathway genes and the relative content of flavonoid biosynthetic pathway metabolites clearly explained yellow seed color formation in *B. rapa*. This study provides a foundation for further research on the molecular mechanism of seed coat color formation.

## Introduction

*Brassica rapa* (AA = 20), an original parent species of *Brassica napus* (AACC = 38) and *Brassica juncea* (AABB = 36), is a major rapeseed crop in the Tibetan Plateau and northern part of China with a short growth period and with resistance to barren field. Its seed coat color can be divided into two categories, brown/black and yellow. Yellow seed coat color confers higher oil quality, higher protein content and lower fiber contents, and thus is considered a desirable trait for breeding new hybrids of *B. rapa*, *B. napus* and *B. juncea* (Li et al., 2012; Wang et al., 2016).

Seed coat color formation is mainly due to the accumulation of proanthocyanidins (PAs), the end product of the flavonoid biosynthetic pathway, which are mainly deposited in the innermost cell layer of the testa (chalaza, micropyle and endothelium) (Debeaujon et al., 2003; Dixon, Xie & Sharma, 2005; Lepiniec et al., 2006). Different results of many studies on the seed coat color of *Brassica* species showed that seed coat color was controlled by different transcription factors and structural genes, such as *TT10* (Fu et al., 2007), *TT8* (Li et al., 2012), *TT2* (Zhou et al., 2016), *TT6* (Huang et al., 2016), *TT1* (Wang et al., 2016) and *TTG1* (Ren et al., 2017).

Genes involved in the flavonoids biosynthetic pathway are divided into two categories, early biosynthetic genes (EBGs) (i.e., *naringenin-chalcone synthase* (*CHS*), *chalcone isomerase* (*CHI*), *naringenin 3-dioxygenase* (*F3H*) and *flavonoid 3′-monooxygenase* (*F3′H*)) involved in the production of common precursors; and late biosynthetic genes (LBGs), which are the downstream genes of the pathway (i.e., *dihydrokaempferol 4-reductase* (*DFR*), *leucocyanidin oxygenase* (*LDOX*) and *anthocyanidin oxidoreductase* (*BAN*)) (Pelletier, Burbulis & Winkel-Shirley, 1999; Lepiniec et al., 2006; Xu, Dubos & Lepiniec, 2015). However, the downstream gene *flavonol synthase* (*FLS*), is also considered to be an EBG. In addition to these structural genes, some regulatory genes, including *TT8*, *TTG1*, *TT1*, *TT2* and *TT10*, are potentially that possibly responsible for yellow seed color in *Brassica* species (Fu et al., 2007; Li et al., 2012; Padmaja et al., 2014; Huang et al., 2016; Wang et al., 2016; Zhou et al., 2016; Wang et al., 2017). Zhao et al. (2019) identified three transcription factors, Bra028039 (*NAC*), Bra023223 (*C2H2 type zinc finger*) and Bra032362 (*TIFY*) as possible candidates involved in regulation of seed coat color in the *B. rapa* varity Yellow Sarson.

Flavonoid biosynthesis genes and regulatory genes are involved in seed coat color and PAs formation. Qu et al. (2016) analyzed the expression of flavonoid biosynthesis genes in six yellow- and black-seeded *B. napus* inbred lines with different genetic background and found that three structural genes (*BnTT3*, *BnTT18* and *BnBAN*), two regulatory genes (*BnTTG2* and *BnTT16*) and three genes encoding transfer proteins (*BnTT12*, *BnTT19* and *BnAHA10*) might play crucial roles in the formation of different seed coat colors. Hong et al. (2017) also found that down-regulation of 12 biosynthetic genes and three regulatory genes involved in the flavonoid pathway mainly contributed to the reduction of PAs in yellow seed coats. Qu et al. (2020) confirmed that *BnTT3*, *BnTT18*, *BnTT10*, *BnTT12* and *BnBAN* involved in the PA pathway had lower expression levels in yellow-seeded rapeseed, strongly suggesting that seed coat color is mainly determined by levels of epicatechin and its derivatives. Zhai et al. (2020) proved that *BnTT8* is vital for seed color by creating yellow-seeded mutants in rapeseed using CRISPR/Cas9. In *B. juncea*, Liu et al. (2013, 2016) found that *BjuDFR, BjuLDOX* and *BjuANR, BjuTT3*, *BjuTT18*, *BjuTT4-2*, *BjuTT4-3*, *BjuTT19-1* and *BjuTT19-3* were transcriptionally regulated, and either not expressed or down-regulated in yellow-seeded testa, implying that these genes for PA biosynthesis are associated with seed color.

Although many studies have reported transcript differences between yellow and brown seeds of *B. napus* and *B. juncea*, there are few studies on yellow and brown seeds coats development using in *B. rapa* using RNA-sequencing (RNA-seq) technology. Previous studies have focused on oil accumulation (Wan et al., 2017), fatty acid degradation, nutritional functions, lipid biosynthesis (Chen et al., 2015a) and seed oil content (Huang et al., 2017). As a method of revealing gene expression levels through high-throughput sequencing techniques, RNA-seq is an effective approach for studying the mechanisms underlying metabolite variation. Nevertheless, it is still difficult to relate phenotypic traits to transcript abundance. Thus, we analyzed the color component of seed coats by liquid chromatography-tandem mass spectrometry (LC-MS/MS). By combining transcript abundance from RNA-seq with color components in the seed coats of brown and yellow seeds, it was possible to determine the genes related to seed coat color variation.

To investigate the genes involved in seed coat color formation in *B. rapa*, we used seeds of the brown-seeded inbred line B147 and its yellow-seeded near-isogenic line B80, at 10, 14 and 28 days after flowering (DAF), each with three biological replicates to construct 18 libraries for analysis of RNA-Seq and for LC-MS. We performed comprehensive analysis of abundant transcripts associated with seed coat color and corresponding flavonoid compounds. Results obtained in this study will provide transcript information for elucidating the molecular mechanism underlying seed coat formation in *Brassica* species.

## Materials and Methods

### Plant materials

**Figure 1:**
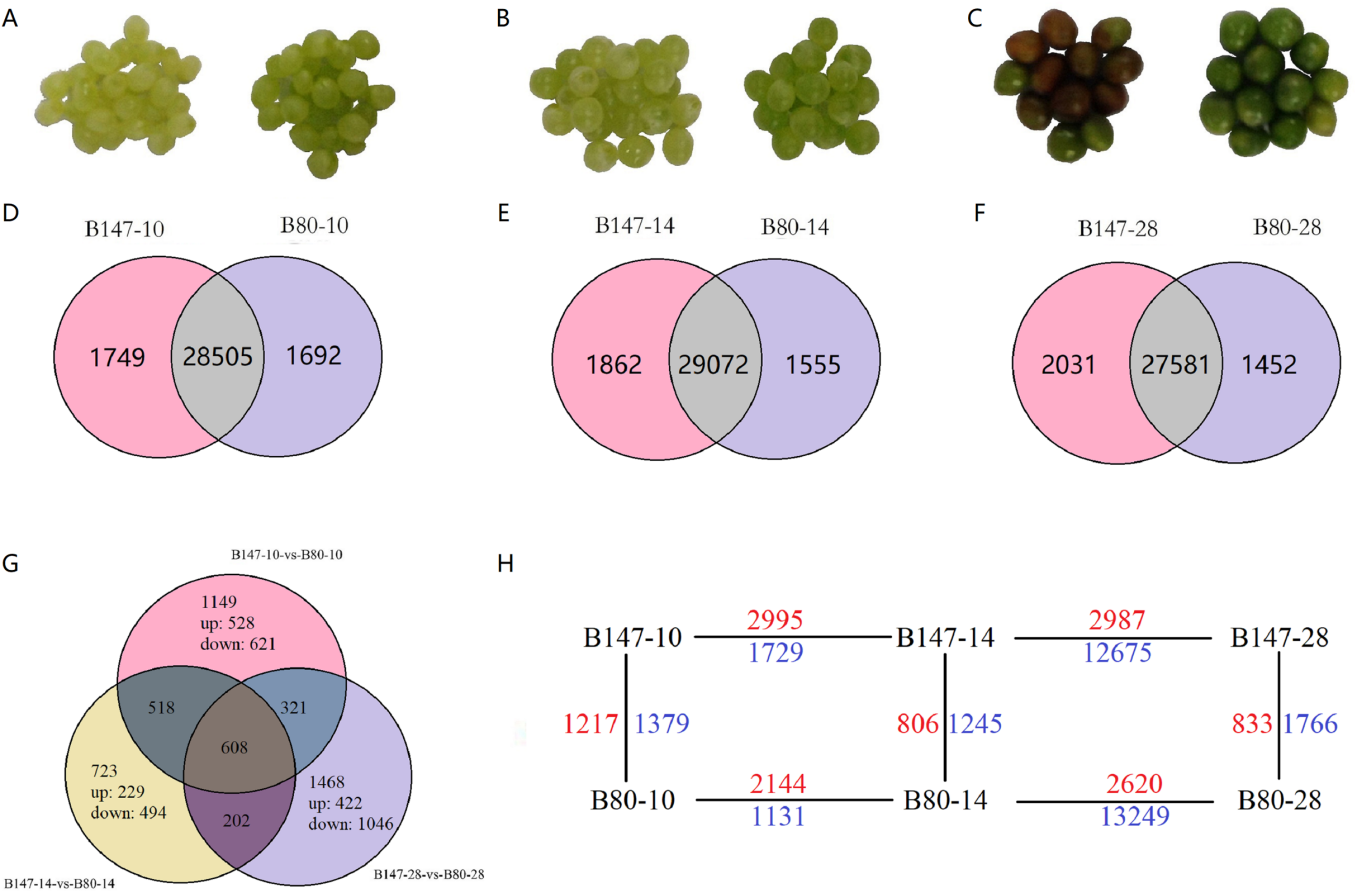
Differentially expressed genes (DEGs) analysis. (A)–(C) Seeds of B147 and B80 at 10, 14 and 28 DAF. (D–F) Venn diagram of genes expressed between each developmental stage of B147 and B80. (G) Venn diagram of genes expressed in pairwise comparisons of B147-10 vs. B80-10, B147-14 vs. B80-14, and B147-28 vs. B80-28. (H) DEGs in multiple pairwise comparison. While green numbers represent down-regulated genes should be while blue numbers represent down-regulated genes.

Two *B. rapa* lines, the brown-seeded inbred line B147 and its yellow-seeded near-isogenic line B80, were provided by the Cruciferae Vegetable Breeding Team at Northwest A&F University, planted at the experimental station of Northwest A&F University, Yangling, China. Fresh seeds were collected at 10, 14 and 28 DAF by artificial pollination at the flowering stage and stored at −80 °C for RNA extraction and flavonoid components analysis (Figs. 1A–1C).

### RNA extraction, library construction and illumina sequencing

Total RNA from each sample was extracted using TRIzol Reagent (Life technologies, Carlsbad, CA, USA) following the manufacturer’s instructions, qualified using a NanoDrop 2000 spectrophotometer (NanoDrop Technologies, Wilmington, DE, USA) and then assessed for quality using an Agilent 2100 Bioanalyzer (Santa Clara, CA, USA). Eukaryotic mRNA was enriched from total RNA using oligo (dT) beads, while prokaryotic mRNA was enriched by removing ribosomal RNA (rRNA) using a Ribo-Zero™ Magnetic Kit (Epicentre). The enriched mRNA was fragmented into short fragments using a fragmentation buffer and reverse transcribed into cDNA using random primers. Second-strand cDNA was synthesized using DNA polymerase I, RNase H, dNTPs and buffer. CDNA fragments were purified using a QiaQuick PCR extraction kit prior to end repair, poly (A) addition, and ligation to Illumina sequencing adapters. The ligation products were size selected by agarose gel electrophoresis, PCR amplified, and sequenced using an Illumina HiSeqTM 2500 by Gene Denovo Biotechnology Co. (Guangzhou, China) (Zhao et al., 2020).

### Transcriptome data processing

High-quality clean reads were obtained by removing adapter reads, reads containing more than 10% of unknown nucleotides (N) and low-quality reads containing more than 50% of low-quality (*Q*-value ≤ 20) bases. Reads mapping to rRNA, detected by the short reads alignment tool Bowtie2 (Langmead & Salzberg, 2012), were removed, and the remaining reads for each sample were mapped to the reference genome (http://Brassicadb.org/brad/) by TopHat2 (Kim et al., 2013), respectively. Alignment parameters were as follows: maximum read mismatch, 2; distance between mate-pair reads, 50bp; error of distance between mate-pair reads, ±80 bp. During the last step of assembly, all of the reassembled fragments were aligned with reference genes and similar fragments were removed.

### Gene annotations, classification and enrichment analysis

Gene expression level was normalized using the FPKM (Fragments Per Kilobase of transcript per Million mapped reads) method and calculated using the following formula: FPKM = (10^6^ × C × 10^3^)/NL, where C is the number of fragments mapped to a specific unigene, N is the total number of fragments mapped to reference genes, and L is the number of bases in this unigene. The FPKM method is able to eliminate the influence of different gene lengths and sequencing data amounts on the calculation of gene expression. The edgeR package (http://www.rproject.org/) was used to identify differentially expressed genes (DEGs) across samples. Genes with a fold change ≥2 and a false discovery rate (FDR) < 0.05 in a comparison were identified as significant DEGs. DEGs were then subjected to enrichment analysis of GO functions and Kyoto Encyclopedia of Genes and Genomes (KEGG) pathways.

Calculated *P*-values were subjected to FDR correction, taking FDR ≤ 0.05 as a threshold. GO terms meeting this condition were defined as significantly enriched GO terms among DEGs. This analysis was able to recognize the main biological functions that DEGs exercise. KEGG enrichment analysis identified metabolic pathways or signal transduction pathways significantly enriched with DEGs, comparing with the whole genome background. The formula for calculation was the same as that in GO analysis. Similarly, the calculated *P*-value was subjected to FDR correction, taking FDR ≤ 0.05 as a threshold. Pathways meeting this condition were defined as pathways significantly enriched in DEGs.

### Weighted gene co-expression network analysis

Weighted gene co-expression network analysis (WGCNA) is a typical systematic biological arithmetic for describing the gene co-expression network of multiple samples and was based on the data of *Brassica rapa* in the STRING protein interaction database by using the interaction relationships (Langfelder & Horvath, 2008; Cheng, Ping & Chen, 2017). It can not only cluster highly correlated genes modules and generalize such modules by the module eigengene or an intramodular hub gene, but also relate modules to one another and to external sample traits (Langfelder & Horvath, 2008). WGCNA was used to analyze the correlation between the transcription factor *TTG1*, the candidate gene for seed coat color (Ren et al., 2017), and those of structural genes in the flavonoid biosynthetic pathway.

### Reverse-transcription quantitative PCR analysis

Reverse-transcription quantitative PCR (RT-qPCR) was conducted to verify the reliability of the transcriptome data. Specific PCR primers were designed using Primer Premier 5. Total RNA was extracted as described above. First-strand cDNA synthesis was performed using a PrimeScript™ RT reagent Kit (TaKaRa, Tokyo, Japan) following the manufacturer’s instructions, and the final cDNA concentration was adjusted to 100 ng/μL with RNase free water. RT-qPCR was performed in 20 μL volume in a QuantStudio 5 Real-Time PCR Detection System with three replications, according to previous reports (Ren et al., 2017); a housekeeping gene encoding glyceraldehyde-3-phosphate dehydrogenase (GAPDH, GO0048316) was used as an internal control (Li et al., 2016). The relative expression levels of genes were measured using the 2^−ΔΔCt^ method (Livak & Schmittgen, 2001).

### Measurement of seed flavonoids, reagents and standards

Extraction of seed flavonoids was performed as described in a previous report (Qu et al., 2013) with minor modification. Frozen fresh pulverous seed samples (0.2 g fresh weight) were soaked in 1.3 mL 75% methanol, and the suspension was placed in an ultrasonic bath for 1 h (Qu et al., 2013). The extract was then centrifuged (12,000 rpm, 20 min) and the limpid solution was collected. The extract was filtered through a 0.22 μm filtration membrane and immediately subjected to LC-MS analysis. Liquid chromatography separations were performed using an XTerra MS C18 column (125 Å pore size, 5 μm, 150 mm × 20 mm; Waters, Milford, MA, USA). The mobile phase consisted of water containing 0.1% (v/v) formic acid (A) and acetonitrile (B) using the following binary gradient: 0–3 min, isocratic 100% A; 3–7 min, isocratic 95% A and 5% B; 7–12 min, isocratic 10% B; 12–19 min, isocratic 25% B; 19–30 min, isocratic 45% B; 30–42 min, isocratic 60% B; 42–50 min, isocratic 95% A and 5% B. The flow rate was 1.2 ml min^–1^ and the temperature of the column was maintained at 25 °C. Samples were analyzed using an LTQ XL linear ion trap mass spectrometer (Thermo, Wilmington, MA, USA) with an ion source voltage of 3.5 kV, a counter current nitrogen flow set at a pressure of 12 psi, and a capillary temperature of 350 °C. Mass spectra were recorded over the range 75–2,000 m/z. Tandem mass spectra were obtained in manual mode for targeted masses using an isolation width of 2.0, fragmentation amplitude of 2.2, and threshold set at 6,000. Chromatographic pure methanol (Kermel, Qingdao, China) was used for extraction of seed flavonoids, and ultra-pure water was obtained using a MilliQ Reference system (Billerica, MA, USA). Solvents for LC-MS were also chromatography grade. Flavonoid standards, kaempferol, quercetin and epicatechin were from Sigma-Aldrich (St. Louis, MO, USA).

## Results

### RNA sequencing and assembly

To explain the molecular basis of seed coat color formation in developing seeds *of B. rapa*, we used seeds from brown-seeded (B147) and yellow-seeded (B80) lines at 10, 14 and 28 DAF with three biological replicates each, to build 18 libraries for sequencing by Illumina RNA-seq. Between 4,004.21 and 5,871.15 Mbytes of raw data and 3,969.87 and 5,815.16 Mbytes of high-quality clean data were generated from the 18 libraries, with Q20 percentages ranging from 97.50% to 98.13%, Q30 percentages ranging from 94.24% to 95.46%, and GC content ranging from 46.91% to 49.75% (Table S1). The filtered clean data were assembled into clean reads. The number of clean reads ranged from 30,086,000 to 34,020,000. After removal of rRNA, an average of 73.74% reads were mapped to the reference database (http://Brassicadb.org/brad/) (Table S2). Finally, a total of 41,286 unigenes were identified from the 18 libraries of B147 and B80 at 10, 14 and 28 DAF (Online Addition File 1). Gene annotations were provided by BLASTx alignment with a threshold *E*-value of 1.0E−05, and genes were matched with sequences in the NCBI non-redundant Protein (NR), Swiss-Prot and KEGG database, respectively.

### Identification of DEGs in seeds at different developmental stages and enrichment analysis

Transcript abundances were quantified using the software RSEM (Li & Dewey, 2011) and expression levels of genes were calculated using the FPKM method. DEGs across samples were estimated using the edgeR package (http://www.rproject.org/). A total of 4,989 DEGs by multiple comparisons between B147 and B80 at the same developmental period were identified (Online Addition File 2). To identify DEGs between B147 and B80, we performed multiple comparisons of DEGs between B147 and B80 at the diverse development stage of 10, 14 and 28 DAF (Figs. 1D–1F). We identified large numbers of DEGs between B147 and B80; 2,596 DEGs (1,217 up-regulated and 1,379 down-regulated) at 10 DAF, 2051 DEGs (806 up-regulated and 1,245 down-regulated) at 14 DAF and 2,599 DEGs (833 up-regulated and 1,766 down-regulated) at 28 DAF. After identifying the DEGs in pairwise comparisons of B147-10 vs. B80-10, B147-14 vs. B80-14 and B147-28 vs. B80-28, we next identified 608 common DEGs in all three pairwise comparisons (Fig. 1G). The DEGs between all the pairwise comparisons were showed in the Fig. 1H.

To better classify the biological functions of these DEGs, we used GO enrichment analysis to identify all GO terms significantly enriched among DEGs compared to the genome background and to filter the DEGs according to their corresponding biological functions. DEGs at 10 DAF were classified into 41 GO categories including 18 biological processes, 14 cellular components and nine molecular functions; DEGs at 14 DAF were classified into 40 GO categories comprising 18 biological processes, 14 cellular components and eight molecular functions and DEGs at 28 DAF were also categorized into 40 GO categories comprising 18 biological processes, 14 cellular components and eight molecular functions. The GO term ‘transcription factor activity, protein binding’ (GO:0000988) in the molecular function category was unique at 10 DAF. In the biological process category, ‘metabolic process’ (GO:0008152), ‘cellular process’ (GO:0009987) and ‘single-organism process’ (GO:0044699) were the most abundant subgroups while ‘biological adhesion’ (GO:0022610), ‘immune system process’ (GO:0002376) and ‘locomotion’ (GO:0040011) comprised the smallest proportion. In the cellular components category, the dominant groups were ‘cell’ (GO:005623), ‘cell part’ (GO:0044464), ‘organelle’ (GO:0043226) and ‘membrane’ (GO:0016020), while ‘extracellular matrix’ (GO:0031012), ‘extracellular region part’ (GO:0044421), ‘cell junction’ (GO:0030054), ‘virion’ (GO:0019012) and ‘virion part’ (GO:0044423) were the least dominant groups. Within the molecular functions category, most DEGs were sorted into ‘binding’ (GO:0005488) and ‘catalytic activity’ (GO:0003824), while the fewest DEGs were sorted into ‘transcription factor activity, protein binding’ (GO:0000988) at 10 DAF, and ‘molecular transducer activity’ (GO:0060089) at 14 and 28 DAF (Online Addition Files 3–5).

To further predict and understand the possible biological pathways involved in seed coat formation, we mapped DEGs to the public pathway-related database KEGG. DEGs between B147 and B80 at 10, 14 and 28 DAF were allocated to 118, 118 and 115 pathways, respectively. Of these, 10, 10 and 9 pathways were significantly enriched (*Q*-value ≤ 0.05, Table S3) in pairwise comparison of B147 and B80 (B147-10 vs. B80-10, B147-14 vs. B80-14 and B147-28 vs. B80-28). These significantly enriched pathways included ‘flavonoid biosynthesis’ (ko00941), ‘phenylpropanoid biosynthesis’ (ko00940), ‘carbon metabolism’ (ko01200) and ‘phenylanlanine metabolism’ (ko00360).

### Analysis of DEGs involved in seed coat color formation

We identified 33 significant DEGs involved in seed coat color pigment formation (Table S4). These DEGs included five copies of *phenylalanine ammonia-lyase* (*PAL*) and five copies of *4-coumarate-CoA ligase* (*4CL*), involved in the phenylpropanoid biosynthetic pathway. The remaining 23 DEGs comprised all copies of *caffeoyl-CoA O-methyltransferase* (*CCoAMT*), *trans-cinnamate 4-monooxygenase* (*C4H*), *naringenin-chalcone synthase* (*CHS*), *chalcone isomerase* (*CHI*), *naringenin 3-dioxygenase* (*F3H*), *flavonoid 3′-monooxygenase* (*F3′H*), *flavonol synthase* (*FLS*), *dihydrokaempferol 4-reductase* (*DFR*), *leucocyanidin oxygenase* (*LDOX*) and *anthocyanidin oxidoreductase* (*BAN*), involved in the flavonoid biosynthetic pathway. *Flavonoid 3′-monooxygenase* (*F3′H*), which is the same as the *F3′H* in the flavonoid biosynthetic pathway, was the only DEG identified in the flavone and flavonol biosynthetic pathway. Among these genes, *CCoAMT* encodes an enzyme converting caffeoyl-CoA into feruloyl-CoA, leading to a final product of homoeriodictyol after two further reactions. The homoeriodictyol does not enter the flavonoid biosynthetic pathway, and therefore, *CCoAMT* is not discussed in this paper.

Based on the FPKM values of these unigenes, different copies of genes showed different expression levels, possibly indicating that these different copies possess different biological functions. Different expression levels of five copies of *PAL* in seeds of B147 and B80 revealed that Bra003126 and Bra005221 possibly have similar functions. Among five copies of *4CL*, the functions of Bra001820 and Bra031263 might be the same; however, transcription levels showed a continuous decline during seed development in B147 and B80, indicating that these two copies show little correlation with seed coat color. Three copies of *C4H*, Bra022803, Bra018311 and Bra021637, might possess a similar function. The functions of three copies on *CHS* are possibly the same on account of the similar transcription levels of these copies in seeds of B147 and B80. Among three copies of *CHI*, the functions of Bra007142 and Bra007145 could be the same. The functions of two copies of *FLS* are likely different because of the significantly different transcription levels of these two copies in seeds of B147 and B80. The functions of two copies of *LDOX* and two copies of *BAN* might be the same on account of their similar transcription levels in seeds of B147 and B80.

**Figure 2:**
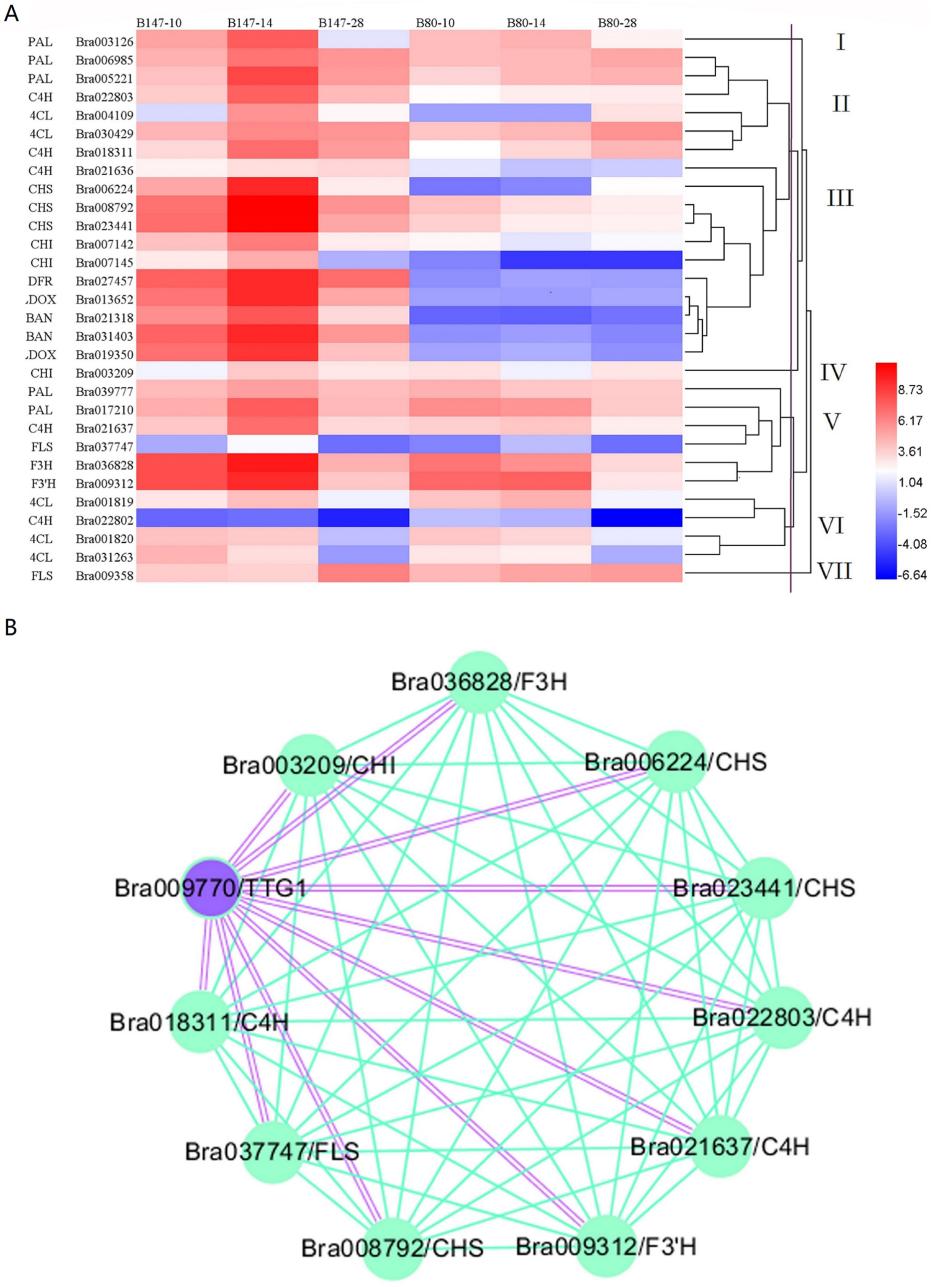
Cluster heatmap of 30 DEGs between B147 and B80, and WGCNA map between *TTG1* and structural genes. (A) Cluster heatmap of 30 DEGs between B147 and B80 at 10, 14 and 28 DAF. (B) WGCNA network analysis map between *TTG1* and structural genes. Purple double lines indicate interactions between transcription factor *TTG1* and structural genes.

We generated a heatmap of transcript expression levels for these 30 genes using the log_2_FPKM values (Fig. 2A). Based on their different transcription levels, these 30 genes were clustered into seven groups, I to VII. Of these seven groups, three contained only one gene, *PAL* (Bra003216) in group I, *CHI* (Bra003209) in group IV and *FLS* (Bra009358) in group VII. The remaining 27 genes were clustered into four groups. Group II contained six genes: *PAL* (Bra006985 and Bra005221), *C4H* (Bra022803 and Bra018311) and *4CL* (Bra004109 and Bra030429). All six of these genes are involved in the phenylpropanoid biosynthetic pathway and had the transcript expression levels that increased from 10 to 14 DAF and decreased from 14 to 28 DAF in seeds of B147, but increased from 10 to 28 DAF in seeds of B80 at a low transcription level. Group III contained 11 genes which were divided into four sub-groups, IIIa, IIIb, IIIc and IIId. Group IIIa and group IIIb consisted of one gene, *C4H* (Bra021636) and *CHS* (Bra006224), respectively. Group IIIc consisted of four genes, *CHS* (Bra008792 and Bra023441) and *CHI* (Bra007142 and Bra007145), which are the EBGs involved in the flavonoid biosynthetic pathway. Transcripts expression levels of these four genes increased from 10 to 14 DAF and decreased from 14 to 28 DAF in seeds of B147, but decreased from 10 to 28 DAF in seeds of B80 at a low transcription level. Group IIId consisted of five genes, *DFR* (Bra027457), *LDOX* (Bra013652 and Bra019350) and *BAN* (Bra021318 and Bra031403), which are LBGs involved in the flavonoid biosynthetic pathway. The transcript expression levels of these five genes increased from 10 to 14 DAF and decreased from 14 to 28 DAF in seeds of B147, but showed little expression in seeds of B80. Group V contained six genes: *PAL* (Bra039777 and Bra017210), *C4H* (Bra021637), *FLS* (Bra037747), *F3H* (Bra036828) and *F3′H* (Bra009312). The transcript expression levels of these six genes increased from 10 to 14 DAF and decreased from 14 to 28 DAF in seeds of B147. In seeds of B80, the transcript levels of these six genes showed no regular expression pattern from 10 to 14 DAF, but expression levels were higher than those in seeds at 28 DAF. Group VI consisted of four genes: *4CL* (Bra001819, Bra001820 and Bra031263) and *C4H* (Bra022802). The transcript expression levels of these four genes showed irregular expression patterns in seeds of B147 and B80, indicating that these four genes were likely not related to the formation of seed coat color. Comprehensive analysis of these seven groups revealed that genes in Group III had a meaningful relationship with the seed coat color formation.

### WGCNA of DEGs involved in the seed coat color with TTG1

Based on a previous study (Ren et al., 2017), we considered the transcription factor *TTG1* to be the candidate gene controlling seed coat color. Hence, WGCNA was used to analyze the association between DEGs involved in seed coat color and *TTG1*. A WGCNA map showed that 10 genes were co-expressed with *TTG1* (Fig. 2B), including *C4H* (Bra022803, Bra018311 and Bra021637), *CHS* (Bra023411, Bra008792 and Bra006224), *CHI* (Bra003209), *F3H* (Bra036828), *F3′H* (Bra009312) and *FLS* (Bra037747). All these structural genes are EBGs in the flavonoid biosynthetic pathway. The closed orbicular WGCNA map with complex interactions between genes indicated that all these structural genes were highly correlated. Three copies of *C4H* and three copies of *CHS* were clustered together in the WGCNA map, suggesting that the three copies of *C4H*, Bra022803, Bra018311and Bra021637, and the three copies of *CHS*, Bra023411, Bra008792 and Bra006224, have a synergic relationship. All these structural genes were clustered into different groups (II, III, IV, V), which indicated that *TTG1* was not the only one transcription factor regulating these genes.

### Structural genes involved in seed coat color

Based on different numbers of gene copies involved in seed coat color formation, the heatmap of transcript expression levels for 30 DEGs and the WGCNA map, 19 structural genes were speculated to be involved in seed coat color formation (Table S5). These genes included three copies of *PAL* (Bra003126, Bra005221 and Bra018311), one copy of *4CL* (Bra004109), two copies of *C4H* (Bra022803 and Bra021637), three copies of *CHS* (Bra006224, Bra008792 and Bra023441), two copies of *CHI* (Bra007142 and Bra007145), *F3H* (Bra036828), *F3′H* (Bra009312), one copy of *FLS* (Bra037747), one copy of *DFR* (Bra027457), two copies of *LDOX* (Bra013652 and Bra019350) and two copies of *BAN* (Bra021318 and Bra031403).

### Transcription factors involved in seed coat color

**Figure 3:**
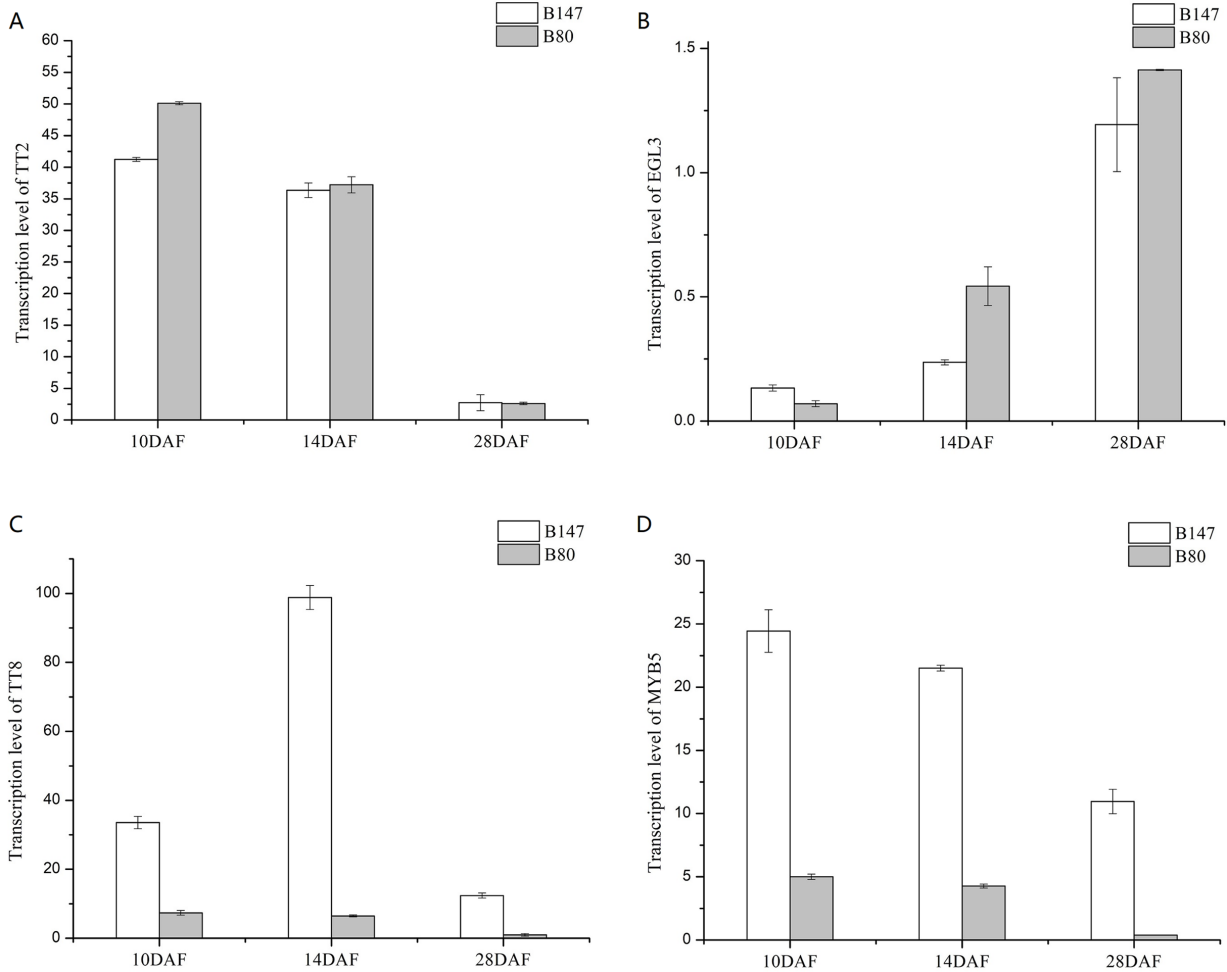
Transcription levels of four transcription factors (*TT2*, *EGL3*, *TT8* and *MYB5*) in seeds of B147 and B80 at 10 DAF, 14 DAF and 28 DAF. (A) *TT2*; (B) *EGL3*; (C) *TT8*; (D) *MYB5*.

We analyzed transcription levels of four transcription factors (*BrTT2*, *BrEGL3*, *BrTT8* and *BrMYB5*) in seeds of B147 and B80 at different developmental stages (10, 14 and 28 DAF) (Fig. 3). Transcription levels of *BrTT2* and *BrEGL3* at 10, 14 and 28 DAF showed no significant difference between B147 and B80. The transcription level of *BrTT2* showed a continuous decline from 10 DAF to 28 DAF, while that of *BrEGL3* showed a continuous increase. The transcription levels of *BrTT8* and *BrMYB5* at 10, 14 and 28 DAF were four-fold higher in B147 than those in B80. In combination with the results of a previous paper (Ren et al., 2017), we propose that a truncated TTG1 without WD40 structures could affect the transcription levels of *TT8* and *MYB5*.

### Expression analysis by RT-qPCR

**Figure 4:**
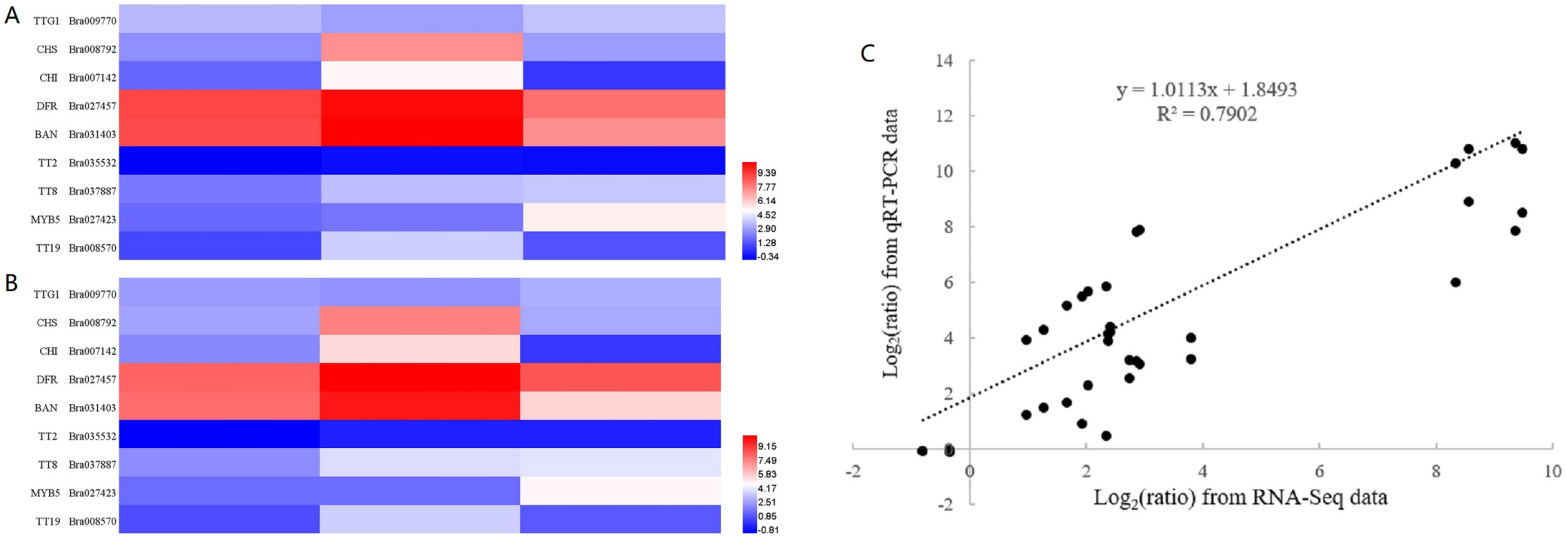
Validation of RNA-seq data by RT-qPCR. (A) Heatmap of the fold change in expression level of nine genes at 10, 14 and 28 DAF with reference to B80 using RNA-Seq. (B) Heatmap of the the fold change in expression level of nine genes at 10, 14 and 28 DAF with reference to B80 using RT-qPCR. (C) The correlation between RNA-Seq and qPCR of the fold change in expression levels of the nine unigenes.

To confirm the validity of RNA-seq data, we selected nine genes, representing transcription factors (*TTG1*, *TT2*, *TT8* and *MYB5*), structural genes involved in the flavonoid biosynthetic pathway (*CHS*, *CHI*, *DFR* and *BAN*) and a gene encoding glutathione-S-transferase (*TT19)*, for RT-qPCR analysis. The sequences of RT-qPCR primers are listed in Table S6. We analyzed the fold change in expression level of these genes at 10, 14 and 28 DAF with reference to B80 were analyzed (Fig. 4). Linear regression analysis indicated that the fold-change in gene expression ratios measured by RT-qPCR and RNA-Seq data were significantly positively correlated (*R*^2^ = 0.7902), which suggested that the transcriptome data were reliable.

### Flavonoids accumulation in seeds of B147 and B80

To detect the difference in flavonoid compounds between B147 and B80, we analyzed seeds at 10, 14 and 28 DAF using LC-MS. Referencing a previous report (Qu et al., 2013), we used three types of flavonoid standards, epicatechin, kaempferol and quercetin, and detected their corresponding flavonol derivatives. Identification of flavonoid compounds was determined as in the previous studies (Qu et al., 2013; Ma et al., 2014; Chen et al. 2015b; De Camargo et al., 2017; Pelvan et al., 2018; Rahman, De Camargo & Shahidi, 2018). We detected 31 flavonoid compounds in B147 and 21 compounds in B80. These flavonoid compounds were divided into two groups, those compounds detected both in B147 and B80, and compounds detected only in B147 (Table 1). Compounds were numbered as the order of retention time.

**Table 1:** Main flavonoid compounds identified by LC-MS/MS in seed flavonoids extracts at 10 DAF, 14 DAF and 28 DAF of brown-seeded inbred line B147 and yellow-seeded line B80.

No.	RT(min)^a^	[M-H]^b^	MS/MS(m/z)^b^	Putative flavonoid compound^c^	B147-10 area	B80-10 area	B147-14 area	B80-14 area	B147-28 area	B80-28 area
Flavonoid compounds detected both in B147 and B80 Flavonoid compounds detected both in B147 and B80 at 10, 14 and 28DAF										
31	21.50	285	241^d^	Kaempferol	275	556	2707	771	1883	2964
8	14.39	639	593,549,519,477,315	Kaempferol-hexoside-glucoside	25,817	7,462	34,988	6,385	28,204	12,169
23	18.39	447	285	Kaempferol-hexoside	2,836	ND	ND	3368	27133	14492
30	20.16	301	255,221,179^d^	Quercetin	4,221	4,788	18,637	4,337	5,119	10,921
13	16.18	625	463,301	Quercetin-glucoside-rhamnoside	87,391	27,812	113,250	23,266	49,614	22,470
32	37.98	591	547,523	Putative quercetin derivative	81,082	41,479	66,517	24,447	68,529	112,421
15	16.34	753	529,289	unknown	14,994	8,003	47,109	13,084	33,214	8,937
14	16.34	639	477,315	Isorhamnetin dihexoside	19,406	12,170	41,690	11,987	60,648	17,350
22	18.11	557	477,315	Isorhamnetinglucoside-rhamnoside	587,048	409,517	2,112,809	809,135	2,228,422	455,492
1	9.95	386	306,275,259	Putative flavonoid	1,293	ND	4,618	19,875	55,783	9,401
12	16.18	609	429,285	Sophoraflavonolside	2,282	ND	2,200	65,186	63,721	173,957
Flavonoid compounds detected in B147 at 10, 14 and 28DAF, while only detected in B80 at 10 and 14DAF										
20	17.78	463	301	Quercetin-hexoside	103,962	10,829	304,219	21,121	17,212	ND
25	18.40	477	315	Isorhamnetin-pentoside	47,083	17,130	99,163	17,275	34,393	ND
29	19.27	418	301,289	Quercetin glucoside rhamnoside	40,532	3,603	204,247	2,055	3,258	ND
Flavonoid compounds detected in B147 and B80 at 14 and 28DAF										
17	16.93	609	447,429,285	Kaempferol-glycoside-rhamnoside	–	–	2,179	65,432	441,238	874,697
18	17.20	545	432,190	Putative flavonol	–	–	ND	11,119	601,574	845,358
Flavonoid compounds detected in B147 and B80 at 28DAF										
3	12.90	771	609	Kaempferol-3-o-diglucoside-7-o-glucoside	–	–	–	–	395,496	109,167
6	13.82	977	815	Kaempferol sinapoy trihexoside	–	–	–	–	997,384	193,294
10	15.42	561	505,448,190	Unkown	–	–	–	–	ND	18,113
24	18.42	559	432,190	Unkown	–	–	–	–	178,454	156,191
28	19.18	591	547,505	Putative quercetin derivative	–	–	–	–	164,250	87,526
Flavonoid compounds detected only in B147 Flavonoid compounds detected in B147 at 10, 14 and 28DAF										
4	13.47	577	451,425,408,289	Procyanidin dimer	68,283	ND	605,765	ND	106,226	ND
9	14.60	289	245,205,179^d^	Epicatechin	640,174	ND	3,383,741	ND	788,480	ND
16	16.42	423	379,377,355,301	Putative quercetin derivative	2,207	ND	9,213	ND	2,079	ND
2	12.83	447	301	Quercetin deoxyhex	6,970	ND	26,717	ND	3,181	ND
26	18.67	418	301,289	Quercetin glucoside rhamnoside	12,482	ND	96,125	ND	5,387	ND
19	17.32	434	289,301	Putative epicatechin derivative	5,605	ND	53,479	ND	131,911	ND
21	18.07	434	289,307	Putative epicatechin derivative	3,910	ND	39,606	ND	86,598	ND
Flavonoid compounds detected in B147 at 10 and 14DAF										
5	13.58	447	301	Quercetin glucuronside	7,488	ND	64,516	ND	–	–
7	14.12	865	739,695,577	Unknown	5,993	ND	53,713	ND	–	–
Flavonoid compounds detected in B147 at 14 and 28DAF										
11	15.87	577	451,425,408	Procyanidin dimer	–	–	26,278	ND	6,745	ND

**Notes:**

a RT, retention time; measured with an XTerra MS C18 column.

b Obtained with an ion trap mass spectrometer.

c Putative compounds were identified after comparison with standards (d) and references.

d Standard identified. ND, none detected; others were detected.

As expected, the three flavonoid compounds (epicatechin, kaempferol and quercetin) and their derivatives were all detected in three seed samples of B147 at three different developmental stages; however, only two compounds and their derivatives (kaempferol and quercetin) were detected in seed samples of B80. No epicatechin, epicatechin derivatives or PAs were found in seeds samples of B80 at any of the three developmental stages, confirming the significant roles of epicatechin and PAs in brown seed coat color formation. Interestingly, PAs with different polymerized forms were found in seeds of B147 at 28 DAF, demonstrating that PA content gradually increased during the process of seed maturation from 14 DAF to 28 DAF.

**Figure 5:**
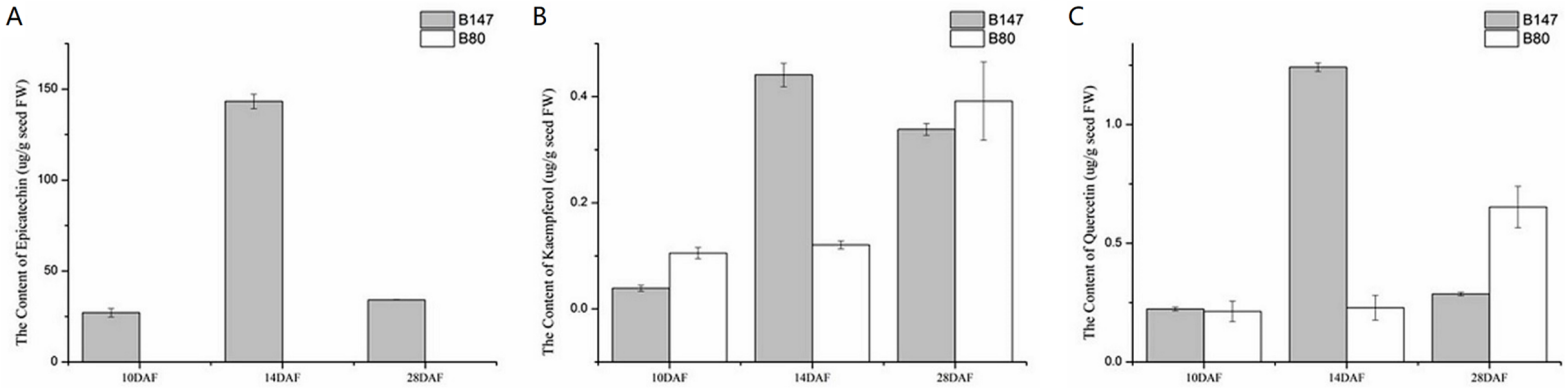
Content of three flavonoid standards, epicatechin, kaempferol and quercetin, in B147 and B80. (A) Content of epicatechin in B147 and B80 at 10, 14 and 28 DAF. (B) Content of kaempferol in B147 and B80 at 10, 14 and 28 DAF. (C) Content of quercetin in B147 and B80 at 10, 14 and 28 DAF.

We examined the content of three flavonoid standards, epicatechin, kaempferol and quercetin in seeds samples of B147 and B80 at 10, 14 and 28 DAF (Fig. 5). The content of these compounds in seeds showed an upward trend from 10 to 14 DAF and a downward trend from 14 to 28 DAF in B147. Epicatechin, the end product of flavonoid biosynthesis catalyzed by oxidoreductase, was not detected in B80, whereas its content peaked at 143 ± 3.97 µg/g fresh weight (µg/g FW) in seeds of B147 at 14 DAF (Fig. 5A). Kaempferol, which is catalyzed by flavonol synthase, was detected in seeds of both B147 and B80. Its content showed no difference between B147 and B80 at 10 and 28 DAF; however, its content was significantly higher in seeds of B147 (0.44 ± 0.02 µg/g FW) than that in seeds of B80 at 14 DAF (Fig. 5B). Quercetin, which is catalyzed by flavonol synthase, was detected in seeds of both B147 and B80, but its content showed no difference at 10 DAF; in seeds at 14 DAF, the quercetin content of B147 (1.24 ± 0.02 µg/g FW) was significantly higher than that of B80; in seeds at 28 DAF, the quercetin content of B147 was lower than that of B80 (Fig. 5C).

### Regulation of yellow seed coat color variation

At the first dedicated step of the flavonoid biosynthetic pathway, except for one unigene (*BrCHI*, Bra003209), expression levels of the other seven genes *BrCHS* (Bra006224, Bra008792 and Bra023441), *BrCHI* (Bra007142, Bra007145), *BrF3H* (Bra036828) and *BrF3’H* (Bra009312) were all higher in seeds of B147 than those of in B80 at 10, 14 and 28 DAF, which indicated that these seven genes are important factors affecting seed coat formation in *B. rapa*. The downstream of flavonoid biosynthesis is divided into two biosynthetic branches. One branch is initiated by BrFLS, an enzyme that converts dihydrokaempferol and dihydroquercetin into kaempferol and quercetin, respectively (Liu et al., 2013). As our results suggested that kaempferol, quercetin and its corresponding derivatives were not important in seed coat color variation, we suppose that BrFLS is independent of seed coat color formation in *B. rapa*. On the other branch, dihydrokaempferol and dihydroquercetin are catalyzed by DFR, LDOX and BAN, successively (Vanderkrol et al., 1990; Boss, Davies & Robinson, 1996; Pelletier, Burbulis & Winkel-Shirley, 1999; Abrahams et al., 2003; Debeaujon et al., 2003; Xie et al., 2003). Transcriptome data showed that these five flavonoid biosynthesis pathway genes were expressed very little or not at all in seeds of B80, and the corresponding flavonoid pathway metabolites, epicatechin, was also not accumulated. We therefore considered that the low expression of these genes resulted in a deficiency of epicatechin and its derivatives, which made the B80 seeds unable to form brown color, resulting in a yellow coat.

**Figure 6:**
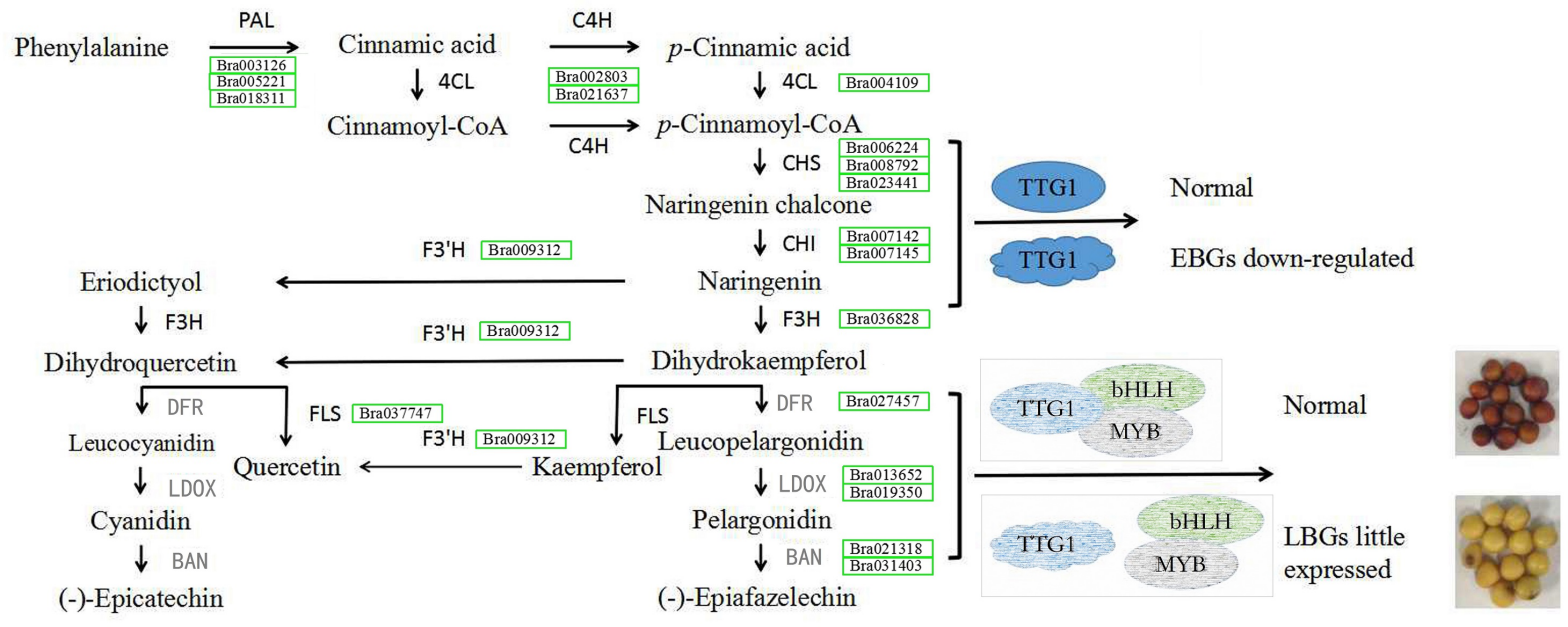
Schematic map of genes involved in seed coat color formation in *B. rapa*. *PAL*, *phenylalanine ammonia-lyase*; *4CL*, *4-coumarate-CoA ligase*; *C4H*, *trans-cinnamate 4-monooxygenase*; *CHS*, *naringenin-chalcone synthase*; *CHI*, *chalcone isomerase*; *F3H*, *naringenin 3-dioxygenase*; *F3′H*, *flavonoid 3′-monooxygenase*; *FLS*, *flavonol synthase*; *DFR*, *dihydrokaempferol 4-reductase*; *LDOX*, *leucocyanidin oxygenase*; *BAN*, *anthocyanidin oxidoreductase*. *DFR*, *LDOX* and *BAN* in grey represent genes with low levels of expression in the yellow-seeded material B80. Green rectangle represents the down-regulated expression level and the gene ID in the green rectangle represents the down-regulated expressed gene in B80. The ellipse represents the normal structure of transcription factor TTG1. The irregular shape represents mutated transcription factor TTG1 without complete WD40 structure. The grey shape indicates the hypothesis for seed coat color formation.

A previous study showed that TTG1 was truncated without WD40 structures in B80 (Ren et al., 2017). We therefore considered that the low expression of *BAN* in seeds of B80 was due to release of binding in the TT2-TT8-TTG1 protein complex. In addition, *DFR*, *LDOX* and *TT8* are direct targets of *TTG1* in Arabidopsis (Gonzalez et al., 2008), which might explain the lower expression of *DFR*, *LDOX* and *TT8* in seeds of B80. In summary, truncated TTG1 without WD40 structures in B80 not only led to the lower expression of *DFR*, *LDOX*, *TT8* and *MYB5*, but also resulted in unbinding of the TT2-TT8-TTG1 protein complex, which could then not induce expression of *BAN*. The low expression of *DFR*, *LDOX* and *BAN* successfully prevented the production of epicatechin, the monomer of PAs, which cause the formation of yellow seeds in *B. rapa* B80 (Fig. 6). In summary, lower expression of six unigenes involving phenylpropanoid biosynthetic pathway, eight EBGs involved in flavonoid biosynthetic pathway and three transcription factors (TTG1, TT2 and MYB5), little or no expression of five LBGs, and release of binding in the TT2-TT8-TTG1 protein complex together regulate the formation of yellow seed coat color in B80 (Fig. 6; Online Addition File 6).

## Discussion

Seed coat color pigments of *Brassica* species are predominantly secondary flavonoid metabolites, that is, flavonols and proanthocyanidins (PAs) (Auger et al., 2010; Fang et al., 2012). Flavonoids are widely distributed in plants ranging from spermatophytes to mosses (Buer, Imin & Djordjevic, 2010; Cheynier et al., 2013; Tohge et al., 2013). PAs are oligomers and polymers of catechin or epicatechin, the end products biosynthesized via the flavonoid biosynthetic pathway (Dixon, Xie & Sharma, 2005; Saito et al., 2013; Xu et al., 2014), and are the main flavonoid compounds affecting the seed coat color in *Brassica* species. In plants, the flavonoid biosynthetic pathway is the second biosynthetic branch from the phenylpropanoid biosynthetic pathway (Wang et al., 2016). Thus, we analyzed significant DEGs involved in the phenylpropanoid, flavonoid, flavone and flavonol biosynthetic pathways related to seed coat color. We determined 19 out of 33 DEGs to be involved in the seed coat formation in B80, because the expression of these 19 genes in B80 was always lower than that in B147.

Xu et al. (2014) reported that the promoters of *CHS*, *CHI* and *F3H* remains unchanged in the seed coat of *ttg1* mutants in *Arabidopsis* seed. Similarly, mRNA accumulation of *CHS*, *CHI* and *F3H* was found in the yellow-seeded line B80, a mutant exhibiting truncated TTG1 without WD40 structures. All these results suggested that the lower transcript expression levels of the above EBGs were affected by a lower transcription level of *TTG1* in seeds of B80. Little expression of LBGs was similar to a report of *B. juncea*, which indicated that these LBGs play crucial roles in the formation of pigment in seed coat in *Brassica* species.

In this study, the peak content of epicatechin appeared in seeds at 14 DAF, which was consistent with the strong expression of *BAN* gene transcript in seeds at 14 DAF. At this stage, large amounts of epicatechin accumulated in seeds and a small quantity was polymerized into PAs. Meanwhile, in seeds at 28 DAF, a large amount of epicatechin was polymerized into PAs, and thus its content was reduced. The variation in content epicatechin from 10 DAF to 28 DAF again confirmed that the period of PA accumulation was 14 DAF. The reduction of kaempferol content in B147seeds at 28 DAF may be due to the formation of kaempferol derivatives via flavone and flavonol biosynthetic pathways and the formation of quercetin catalyzed by flavonoid 3’-monooxygenase. Alternatively, the increased kaempferol content in yellow seeds (B80) at 28 DAF was possibly due to the weak transcript expression level of *F3′H*. Thus, the content of kaempferol appeared to increase from 14 to 28 DAF in yellow seeds of B80. The trend of quercetin content during seed development was consistent with the trend of transcript expression levels of one *FLS* gene, Bra037747. Overall, at least two genes (*FLS*, *F3′H*) together affected the variation in kaempferol and quercetin content.

PAs, the oligomeric and polymeric forms of catechin or epicatechin, are considered to be the main pigments affecting seed coat color in *Brassica* species. However, whether the two other main components in seed coat, kaempferol and quercetin, are related to seed coat color is unknown. We detected the flavonoid compounds kaempferol, quercetin and their corresponding derivatives by LC-MS in both B147 and B80, which indicated that kaempferol, quercetin and their corresponding derivatives are not significant in seed coat color variation.

In our comprehensive analysis of component differences in flavonoids between B147 and B80, epicatechin was unique to B147 at 10, 14 and 28 DAF, indicating that the color difference between brown and yellow seeds of *B. rapa* is due to the loss of epicatechin, the monomeric form of PAs. Many studies have reported that regulation of PAs is due to the combined action of MYB and R/B- like basic helix-loop-helix (bHLH) transcription factors, together with TTG1, in a MYB–bHLH–WDR (MBW) ternary protein complex (Heim et al., 2003; Baudry et al., 2004; Zimmermann et al., 2004; Lepiniec et al., 2006; Dubos et al., 2008; Xu, Dubos & Lepiniec, 2015) and that the TT2-TT8-TTG1 protein complex directly regulates the expression of *BAN* in *Arabidopsis* seed (Baudry et al., 2004). In this study, we found that mutant *TTG1* not only regulates the expression of *TT8* and *MYB5*, but also affects the combination of the MBW complex. All these factors (structural unigenes and transcription factors) together regulate the variation of seed coat color.

MYB–bHLH–WDR complexes formed by transcription factors are known to regulate the seed coat color and WDR has been previously identified, while the identification of the partners, MYB and bHLH members has not been reported in *B. rapa* hitheto. Additional studies on the members of MYB and bHLH families should be continued. The results of this study provide a general framework for future research into the molecular mechanism of seed coat color formation of *Brassica* crops.

## Conclusion

In this study, we used seeds of the brown-seeded inbred line B147, and its yellow-seeded near-isogenic line B80, at 10, 14 and 28 DAF to construct 18 libraries for analysis of RNA-Seq and LC-MS. We identified a total of 41,286 unigenes and 4,989 DEGs by multiple comparisons between B147 and B80 at the same developmental period. KEGG enrichment analysis and WGCNA showed that 19 unigene sequences being associated with the phenylpropanoid, flavonoid, flavone and flavonol biosynthetic pathways were involved in seed coat color formation. Truncated *TTG1* without WD40 structures in B80 not only led to lower expression of *DFR*, *LDOX*, *TT8* and *MYB5*, but also results in unbinding of the TT2–TT8–TTG1 protein complex, which could then not induce the expression of *BAN*. LC-MS also showed that epicatechin, a product at the end of the DFR–LDOX–BAN metabolism flow, was not detected in seeds of B80. These findings will facilitate better understanding of the regulatory mechanism of seed coat color formation in Chinese cabbage.

## Supplemental Information

10.7717/peerj.10770/supp-1 Supplemental Information 1 qRT-PCR raw data.

10.7717/peerj.10770/supp-2 Supplemental Information 2 Seed coat color and multiple comparison between B147 and B80 at 10, 14 and 28 DAF. a. Seed coat color between B147 and B80 at 10, 14 and 28 DAF cited by Ren et al. (2017). b. multiple comparison between B147 and B80 at 10, 14 and 28 DAF. 10 = 10 DAF, 14 = 14 DAF, and 28 = 28 DAF. Red numbers represent the up-regulated genes, while green ones represent the down-regulated genes.

10.7717/peerj.10770/supp-3 Supplemental Information 3 Summary of Illumina transcriptome sequencing of developing seeds in brown-seeded B147 and yellow-seeded B80.

10.7717/peerj.10770/supp-4 Supplemental Information 4 Summary of Illumina transcriptome data assembly analysis of developing seeds in brown-seeded B147 and yellow-seeded B80.

10.7717/peerj.10770/supp-5 Supplemental Information 5 KEGG pathway significant enrichment analysis of differentially expressed genes at at 10, 14 and 28 days between brown-seeded inbred line B147 and its yellow-seed near-isogenic line B80 in *B. rapa*.

10.7717/peerj.10770/supp-6 Supplemental Information 6 The significantly differential expression genes involved in seed coat color pigment formation in *B rapa*.

10.7717/peerj.10770/supp-7 Supplemental Information 7 The summaries of different copies of unigenes involving in seed coat formation.

10.7717/peerj.10770/supp-8 Supplemental Information 8 All primer sequences for reverse-transcription quantitative real-time PCR.

10.7717/peerj.10770/supp-9 Supplemental Information 9 All 41,286 unigenes detected of developing seeds in brown-seeded B147 and yellow-seeded B80.

10.7717/peerj.10770/supp-10 Supplemental Information 10 All 4,989 differentially expressed genes detected of developing seeds in brown-seeded B147 and yellow-seeded B80.

10.7717/peerj.10770/supp-11 Supplemental Information 11 GO terms of differentially expressed genes detected of developing seeds in brown-seeded B147-10 and yellow-seeded B80-10.

10.7717/peerj.10770/supp-12 Supplemental Information 12 GO terms of differentially expressed genes detected of developing seeds in brown-seeded B147-14 and yellow-seeded B80-14.

10.7717/peerj.10770/supp-13 Supplemental Information 13 GO terms of differentially expressed genes detected of developing seeds in brown-seeded B147-28 and yellow-seeded B80-28.

10.7717/peerj.10770/supp-14 Supplemental Information 14 Expression levels of differentially expressed genes involved in the formation of yellow seed coat color.

## Additional Information and Declarations

### Data Availability

The following information was supplied regarding data availability: Data is available at NCBI SRA: SRP128358.
