# Supplementary figures and images for: Comparative transcriptome and flavonoids components analysis reveal the structural genes responsible for the yellow seed coat color of Brassica rapa L
Source: PeerJ. 2021 Mar 4;9:e10770. doi: 10.7717/peerj.10770 (PMC7937345; doi:10.7717/peerj.10770)

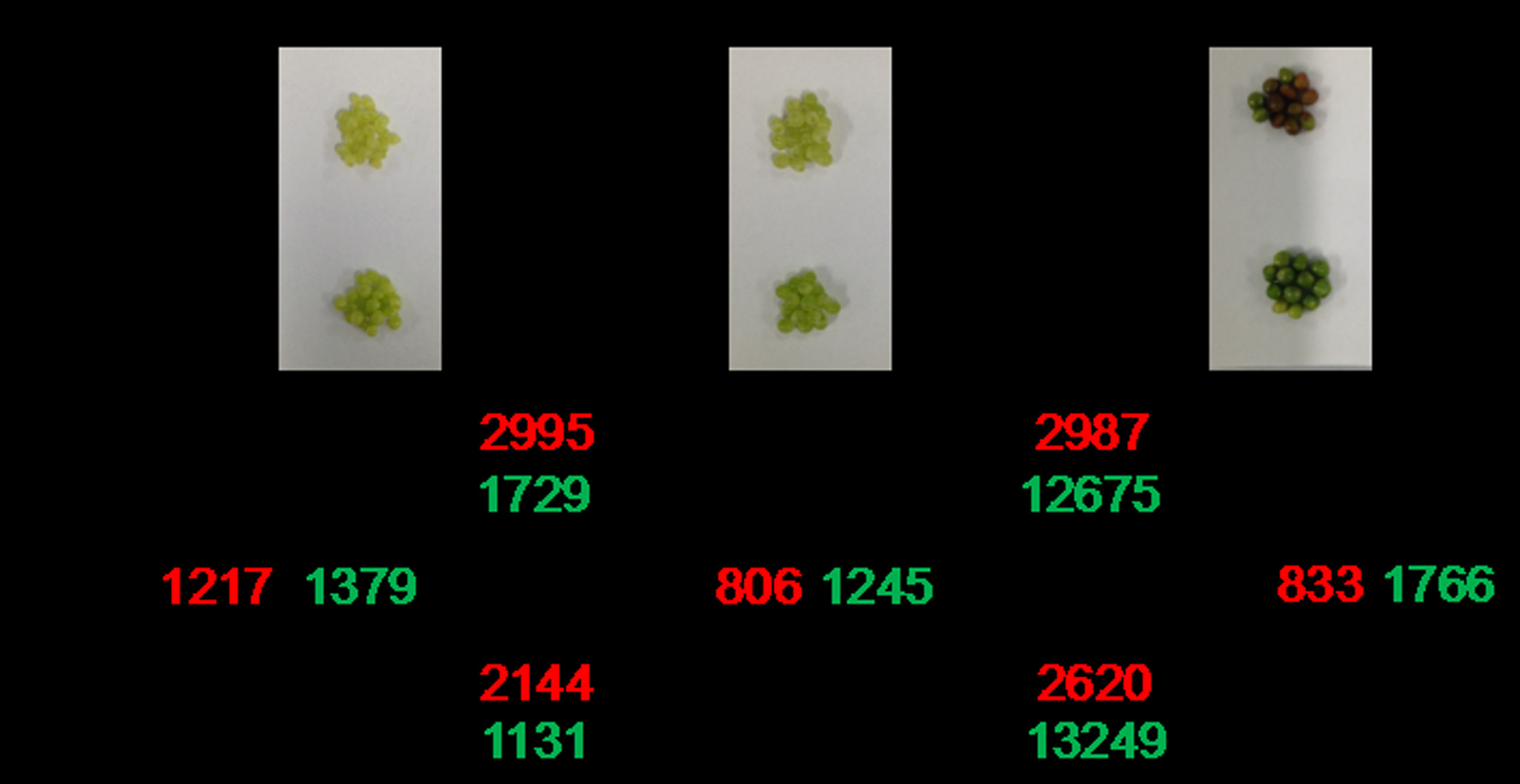

Supplement: Supplemental Information 2 — 10 = 10 DAF, 14 = 14 DAF, and 28 = 28 DAF. Red numbers represent the up-regulated genes, while green ones represent the down-regulated genes. [file peerj-09-10770-s002.jpg]
