# Supplementary material for: Comparative transcriptome and flavonoids components analysis reveal the structural genes responsible for the yellow seed coat color of Brassica rapa L
Source: PeerJ. 2021 Mar 4;9:e10770. doi: 10.7717/peerj.10770 (PMC7937345; doi:10.7717/peerj.10770)
Supplement: Supplemental Information 3 [file peerj-09-10770-s003.doc]

**Table S1. Summary of Illumina transcriptome sequencing of developing seeds in brown-seeded B147 and yellow-seeded B80.**

| Samples | Raw data (Mb) | HQ clean data (Mb) | Q20 | Q30 | GC |
| --- | --- | --- | --- | --- | --- |
| B147-0-10 | 4945.82 | 4905.03 | 97.85% | 94.89% | 47.26% |
| B147-2-10 | 4372.07 | 4338.20 | 98.00% | 95.19% | 46.93% |
| B147-3-10 | 4220.97 | 4188.09 | 97.87% | 94.92% | 47.78% |
| B147-0-14 | 528.157 | 5225.57 | 97.50% | 94.24% | 48.39% |
| B147-2-14 | 4650.97 | 4608.92 | 97.80% | 94.80% | 48.93% |
| B147-3-14 | 5376.87 | 5330.24 | 97.85% | 94.91% | 47.30% |
| B147-0-28 | 4929.33 | 4885.82 | 97.83% | 94.86% | 49.21% |
| B147-2-28 | 5216.62 | 5167.31 | 97.72% | 94.65% | 49.58% |
| B147-3-28 | 4413.71 | 4373.75 | 97.84% | 94.88% | 49.24% |
| B80-0-10 | 4458.92 | 4417.01 | 97.58% | 94.38% | 46.91% |
| B80-4-10 | 4004.21 | 3969.87 | 97.75% | 94.70% | 47.58% |
| B80-5-10 | 5788.81 | 5737.99 | 97.74% | 94.66% | 48.66% |
| B80-0-14 | 4176.12 | 4145.19 | 97.93% | 95.04% | 48.01% |
| B80-4-14 | 5090.90 | 5052.41 | 98.13% | 95.46% | 47.23% |
| B80-5-14 | 5182.03 | 5141.02 | 97.84% | 94.86% | 49.05% |
| B80-0-28 | 5871.15 | 5815.16 | 97.77% | 94.75% | 49.20% |
| B80-4-28 | 4847.29 | 4803.85 | 97.80% | 94.80% | 49.75% |
| B80-5-28 | 4451.53 | 4410.61 | 97.71% | 94.61% | 49.75% |

10 means 10 days after flowering (DAF), 14 = 14DAF and 28 = 28 DAF
