# Supplementary material for: Comparative transcriptome and flavonoids components analysis reveal the structural genes responsible for the yellow seed coat color of Brassica rapa L
Source: PeerJ. 2021 Mar 4;9:e10770. doi: 10.7717/peerj.10770 (PMC7937345; doi:10.7717/peerj.10770)
Supplement: Supplemental Information 4 [file peerj-09-10770-s004.doc]

**Table S2. Summary of Illumina transcriptome data assembly analysis of developing seeds in brown-seeded B147 and yellow-seeded B80.**

| Samples | HQ clean reads number | rRNA unmapped reads | Unique Mapped Reads | Multiple Mapped reads | Mapping Ratio (%) | Genes |
| --- | --- | --- | --- | --- | --- | --- |
| B147-0-10 | 32814 | 32719 (99.71%) | 23382 (71.46%) | 216 (0.66%) | 72.13% | 28060 |
| B147-2-10 | 29027 | 28959 (99.77%) | 20996 (72.50%) | 207 (0.72%) | 73.22% | 28093 |
| B147-3-10 | 28006 | 27935 (99.74%) | 20247 (72.48%) | 192 (0.69%) | 73.17% | 27488 |
| B147-0-14 | 34964 | 34666 (99.15%) | 23898 (68.94%) | 259 (0.75%) | 69.69% | 28261 |
| B147-2-14 | 30842 | 30634 (99.33%) | 21696 (70.82%) | 205 (0.67%) | 71.49% | 27787 |
| B147-3-14 | 35668 | 35536 (99.63%) | 25287 (71.16%) | 254 (0.72%) | 71.88% | 28964 |
| B147-0-28 | 32711 | 32590 (99.63%) | 24392 (74.85%) | 213 (0.66%) | 75.50% | 27515 |
| B147-2-28 | 34594 | 34454 (99.60%) | 26047 (75.60%) | 356 (1.04%) | 76.63% | 26731 |
| B147-3-28 | 29293 | 29198 (99.67%) | 22090 (75.66%) | 330 (1.13%) | 76.79% | 27211 |
| B80-0-10 | 29526 | 29483 (99.85%) | 21068 (71.46%) | 190 (0.65%) | 72.10% | 28043 |
| B80-4-10 | 26543 | 26475 (99.75%) | 19240 (72.67%) | 174 (0.66%) | 73.33% | 27246 |
| B80-5-10 | 38369 | 38239 (99.66%) | 27228 (71.21%) | 243 (0.64%) | 71.84% | 27958 |
| B80-0-14 | 27724 | 27642 (99.71%) | 20072 (72.62%) | 192 (0.70%) | 73.31% | 27911 |
| B80-4-14 | 33819 | 33716 (99.69%) | 24487 (72.63%) | 266 (0.79%) | 73.42% | 28672 |
| B80-5-14 | 34376 | 34258 (99.66%) | 24242 (70.76%) | 260 (0.76%) | 71.52% | 27912 |
| B80-0-28 | 38930 | 38797 (99.66%) | 29679 (76.50%) | 335 (0.87%) | 77.36% | 26803 |
| B80-4-28 | 32147 | 31979 (99.48%) | 24487 (76.57%) | 298 (0.93%) | 77.51% | 26566 |
| B80-5-28 | 29507 | 29354 (99.48%) | 22169 (75.52%) | 274 (0.93%) | 76.46% | 26224 |

10 means 10 days after flowering (DAF), 14 = 14DAF and 28 = 28 DAF
