# Supplementary material for: Comparative transcriptome and flavonoids components analysis reveal the structural genes responsible for the yellow seed coat color of Brassica rapa L
Source: PeerJ. 2021 Mar 4;9:e10770. doi: 10.7717/peerj.10770 (PMC7937345; doi:10.7717/peerj.10770)
Supplement: Supplemental Information 5 [file peerj-09-10770-s005.doc]

**Table S3. KEGG pathway significant enrichment analysis of differentially expressed genes at at 10, 14 and 28 days between brown-seeded inbred line B147 and its yellow-seed near-isogenic line B80 in *B.rapa.***

| Pathway | Number of DEGs | Q-value | Pathway ID |
| --- | --- | --- | --- |
| 10DAF | | | |
| Flavonoid biosynthesis | 17 | 0.000001 | ko00941 |
| Phenylpropanoid biosynthesis | 46 | 0.000003 | ko00940 |
| Carbon metabolism | 57 | 0.000335 | ko01200 |
| Phenylalanine metabolism | 16 | 0.000946 | ko00360 |
| Circadian rhythm - plant | 13 | 0.015049 | ko04712 |
| Carbon fixation in photosynthetic organisms | 19 | 0.015685 | ko00710 |
| Glyoxylate and dicarboxylate metabolism | 20 | 0.016216 | ko00630 |
| Tryptophan metabolism | 14 | 0.035186 | ko00380 |
| Alanine, aspartate and glutamate metabolism | 14 | 0.035186 | ko00250 |
| Cyanoamino acid metabolism | 16 | 0.046352 | ko00460 |
| 14DAF | | | |
| Flavonoid biosynthesis | 20 | 0.000000 | ko00941 |
| Phenylalanine metabolism | 17 | 0.000017 | ko00360 |
| Phenylpropanoid biosynthesis | 35 | 0.000208 | ko00940 |
| Carbon metabolism | 45 | 0.002178 | ko01200 |
| Biosynthesis of amino acids | 40 | 0.009962 | ko01230 |
| Degradation of aromatic compounds | 5 | 0.018178 | ko01220 |
| Valine, leucine and isoleucine degradation | 12 | 0.018178 | ko00280 |
| Phenylalanine, tyrosine and tryptophan biosynthesis | 12 | 0.018178 | ko00400 |
| Propanoate metabolism | 9 | 0.020739 | ko00640 |
| Carbon fixation in photosynthetic organisms | 15 | 0.033408 | ko00710 |
| 28DAF | | | |
| Phenylpropanoid biosynthesis | 54 | 0.000000 | ko00940 |
| Flavonoid biosynthesis | 15 | 0.000074 | ko00941 |
| Carbon fixation in photosynthetic organisms | 25 | 0.000136 | ko00710 |
| Cyanoamino acid metabolism | 21 | 0.001722 | ko00460 |
| Cutin, suberine and wax biosynthesis | 15 | 0.001722 | ko00073 |
| Starch and sucrose metabolism | 47 | 0.002361 | ko00500 |
| Glutathione metabolism | 22 | 0.020241 | ko00480 |
| Fatty acid metabolism | 21 | 0.024539 | ko01212 |
| Carbon metabolism | 50 | 0.049136 | ko01200 |
