# Supplementary material for: Comparative transcriptome and flavonoids components analysis reveal the structural genes responsible for the yellow seed coat color of Brassica rapa L
Source: PeerJ. 2021 Mar 4;9:e10770. doi: 10.7717/peerj.10770 (PMC7937345; doi:10.7717/peerj.10770)
Supplement: Supplemental Information 6 [file peerj-09-10770-s006.doc]

**Table S4. The significantly differential expression genes involved in seed coat color pigment formation in *B rapa*.**

| Unigenes | Gene name | Function annotation |
| --- | --- | --- |
| Bra003126, Bra006985, Bra039777, Bra005221, Bra017210 | PAL | phenylalanine ammonia-lyase |
| Bra004109, Bra001819, Bra001820, Bra031263, Bra030429 | 4CL | 4-coumarate-CoA ligase |
| Bra017624, Bra033968, Bra012494 | CCoAMT | caffeoyl-CoA O-methyltransferase |
| Bra018311, Bra022802, Bra022803, Bra021636, Bra021637, | C4H | trans-cinnamate 4-monooxygenase |
| Bra006224, Bra008792, Bra023441 | CHS | naringenin-chalcone synthase |
| Bra003209, Bra007142, Bra007145 | CHI | chalcone isomerase |
| Bra036828 | F3H | naringenin 3-dioxygenase |
| Bra009312 | F3’H | flavonoid 3'-monooxygenase |
| Bra009358, Bra037747 | FLS | flavonol synthase |
| Bra027457 | DFR | dihydrokaempferol 4-reductase |
| Bra013652, Bra019350 | LDOX | leucocyanidin oxygenase |
| Bra021318, Bra031403 | BAN | oxidoreductase |
