# Supplementary material for: Comparative transcriptome and flavonoids components analysis reveal the structural genes responsible for the yellow seed coat color of Brassica rapa L
Source: PeerJ. 2021 Mar 4;9:e10770. doi: 10.7717/peerj.10770 (PMC7937345; doi:10.7717/peerj.10770)
Supplement: Supplemental Information 7 [file peerj-09-10770-s007.doc]

**Table S5. The summaries of different copies of unigenes involving in seed coat formation.**

| Gene name | The number of copies | Copies involved in seed coat color formation |
| --- | --- | --- |
| PAL | 5 | Bra003126, Bra005221, Bra018311 |
| 4CL | 5 | Bra004109 |
| C4H | 5 | Bra022803, Bra021637 |
| CHS | 3 | Bra006224, Bra008792, Bra023441 |
| CHI | 3 | Bra007142, Bra007145 |
| F3H | 1 | Bra036828 |
| F3’H | 1 | Bra009312 |
| FLS | 2 | Bra037747 |
| DFR | 1 | Bra027457 |
| LDOX | 2 | Bra013652, Bra019350 |
| BAN | 2 | Bra021318, Bra031403 |
