# Supplementary material for: Comparative transcriptome and flavonoids components analysis reveal the structural genes responsible for the yellow seed coat color of Brassica rapa L
Source: PeerJ. 2021 Mar 4;9:e10770. doi: 10.7717/peerj.10770 (PMC7937345; doi:10.7717/peerj.10770)
Supplement: Supplemental Information 8 [file peerj-09-10770-s008.docx]

**Table S6. All primers sequences for quantitative real-time PCR.**

| Primers name | ID | Forwarding sequences 5’-3’ | Reversed sequence 5’-3’ |
| --- | --- | --- | --- |
| GAPDH | GO0048316 | TAACTGCCTTGCTCCACTTGC | CGGTGCTGCTGGGAATGAT |
| TTG1 | Bra009770 | TGTATGGCGACGATTCTGA | CAATCCCATTCGGTCCAG |
| CHS | Bra008792 | CCCATCTGACACCCACCTTG | TCTTCTCCGCCTTGAGCCCT |
| CHI | Bra007142 | GGAGAACTGTGTGGCGATATGG | AGGGGAGAGAGCGAAGAGGATG |
| DFR | Bra027457 | GCTACGATGACGCCATAAAC | TTCCAGCAGACGAAGTAAACAC |
| BAN | Bra031403 | ATCAATCCAGCGATACAAGG | TTCGGTCATCACAAGTCCAG |
| TT2 | Bra035532 | ACCAACCAACAGAAAAGTCG | AACAATGAAGGAGAACCAGC |
| TT8 | Bra037887 | TCTACTCATCACGAGCCAAAC | CGCATCTCATCTCTAACAAAAC |
| MYB5 | Bra027423 | GCAAACAACACCCATAAACCT | TGTCATCGTAACAGAAACCAAA |
| TT19 | Bra008570 | CCAGTGGGCTGATGTTGA | AAACGGCGTCACATTCTT |
